# Bumped Kinase Inhibitors Inhibit both Toxoplasma gondii MAPKL1 and CDPK1

**DOI:** 10.1021/acsinfecdis.5c00051

**Published:** 2025-05-23

**Authors:** Jemma A. Montgomery, P. Holland Alday, Ryan Choi, Monique Khim, Bart L. Staker, Matthew A. Hulverson, Kayode K. Ojo, Erkang Fan, Wesley C. Van Voorhis, J. Stone Doggett

**Affiliations:** † Division of Infectious Diseases, Portland VA Medical Center, Portland, Oregon 97239, United States; ‡ Division of Infectious Diseases, 20088 Oregon Health & Science University, Portland, Oregon 97239, United States; § Center for Emerging and Re-emerging Infectious Diseases (CERID), Division of Allergy and Infectious Diseases, Department of Medicine, 7284 University of Washington, Seattle, Washington 98109, United States; ∥ Seattle Structural Genomics Center for Infectious Disease (SSGCID), Seattle, Washington 98109, United States; ⊥ Center for Global Infectious Disease Research, 145793 Seattle Children’s Research Institute, Seattle, Washington 98109, United States; # Department of Biochemistry, University of Washington, Seattle, Washington 98109, United States

**Keywords:** toxoplasmosis, antiparasitic drugs, bumped kinase inhibitor, drug mechanism, therapeutics, kinase

## Abstract

A subset of bumped kinase inhibitors (BKIs) has been optimized to inhibit the calcium dependent protein kinase 1 (*Tg*CDPK1; TGGT1_301440) of Toxoplasma gondii and pathogenic *Cryptosporidium* species. Extensive preclinical development of BKIs has identified BKI-1748 as a highly effective lead compound for toxoplasmosis. Phenotypic and therapeutic effects of BKIs have suggested that BKIs may have targets other than *Tg*CDPK1. The mechanism of BKI-1748 action was further investigated using a forward genetic screen of chemical mutagenesis, selection of BKI-1748 resistant clones, and whole genome sequence analysis. Resistant clones were found to have single nucleotide changes in the ATP binding site of the T. gondii mitogen-activated protein kinase-like 1 gene (*Tg*MAPKL1; TGGT1_312570). One of the mutations found in the ATP binding pocket, Leu162Gln, was introduced into a wild-type strain, resulting in 2 to 6-fold resistance to BKI-1748 and a set of structurally varied BKIs. A strain with a *Tg*CDPK1 gatekeeper substitution, Gly128Met, was also created and demonstrated a similar degree of resistance. The combination of *Tg*MAPKL1 and *Tg*CDPK1 substitutions together in a single strain resulted in a substantial increase in resistance to BKIs, up to 157-fold. The identification of *Tg*MAPKL1 in addition to *Tg*CDPK1 as a target of BKIs provides a greater understanding of the BKI mechanism of action that is important for further therapeutic development and suggests a high genetic barrier to meaningful drug resistance for this promising class of compounds.

## Graphical abstract

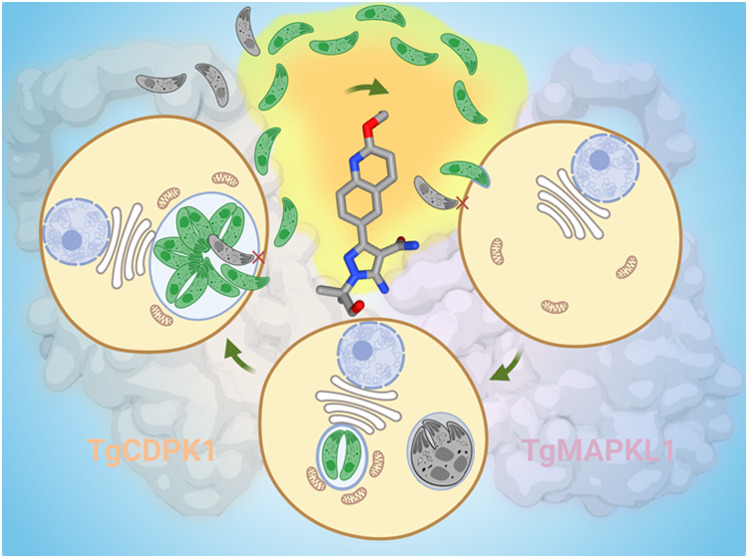

Toxoplasma gondii is a common parasite of humans that can cause debilitating disease or death. The seroprevalence of T. gondii is estimated to be approximately 25–30% worldwide.^1^ T. gondii is transmitted to humans through the ingestion of undercooked meat or as oocysts spread through cat feces.^2^ T. gondii infection leads to multiple disease manifestations with a wide range of severity. Primary T. gondii infection of pregnant women may cause fetal death or severe congenitally acquired disability, however, initial T. gondii infection in nonpregnant immunocompetent individuals often causes a temporary febrile illness or is asymptomatic. After initial infection, the parasite forms dormant tissue cysts that are not eliminated by current treatments or the immune system. Reactivation of these cysts can lead to vision loss in immunocompetent people and can be fatal in immunocompromised patients.^3^

Drugs that have fewer serious adverse effects than current therapy are urgently needed. Bumped Kinase Inhibitors (BKIs) are a promising new class of drugs for toxoplasmosis. BKIs were initially found to inhibit the Calcium Dependent Protein Kinase 1 (*Tg*CDPK1), which is important for parasite motility, invasion and egress from host cells.^4–6^ *Tg*CDPK1 may be selectively targeted because it does not have orthologs in mammals. Furthermore, mammalian kinases generally have a large gatekeeper amino acid residue in the ATP binding pocket, whereas some apicomplexan parasites, such as T. gondii, Cryptosporidium parvum, Sarcocystis neurona, and Neospora caninum, have a smaller glycine gatekeeper residue. This allows the development of inhibitors with a large “bump” that are sterically hindered from binding to mammalian kinases but fit into the larger binding pocket of CDPK1.

Several BKIs are highly effective at reducing parasite burden and have been proven safe in animal models of toxoplasmosis.^7–10^ A promising lead compound, BKI-1748, has been shown to reduce the cerebral parasite load in infected mice and prevent vertical transmission from dam to pups.^9^ Doses of 20 mg/kg/day had no adverse effects on either dam or pup and yielded plasma concentrations of the drug that were 23–67.4 times higher than the T. gondii EC_50_ (50% effective concentration).^9^ In a pregnant sheep model, doses of BKI-1748 at 15 mg/kg administered 48 h after T. gondii infection every 48 h for 10 doses total was sufficient to prevent fetal abortion and vertical transfer of toxoplasmosis to the fetus, compared to 100% fetal mortality in infected but untreated sheep.^8^

Interestingly, treatment of T. gondii with BKIs resulted in large multinucleated complexes, indicating that BKIs may interfere with parasite cell division.^9,11^ Additionally, BKIs have been shown to reduce the number of established brain tissue cysts in mouse models of latent toxoplasmosis wherein mice were treated with BKIs 5 weeks after inoculation and further dissemination of parasites would be expected to be minimal.^12^ Both the multinucleated phenotype and the reduction in tissue cysts after they are established would not be expected from *Tg*CDPK1 inhibition. To identify an alternate unknown target, we used a forward genetic screen of chemical mutagenesis followed by selection with the antitoxoplasmosis lead compound, BKI-1748. The BKI resistance mutations that were identified were then introduced alone and in combination with known *Tg*CDPK1 mutations into a wild-type background and evaluated.

## Results

### Identification of BKI-1748 Resistance Mutations

Three strains of T. gondii were treated with the chemical mutagen, ethylnitrosourea (ENU), at a concentration that resulted in (70%) T. gondii death. Each mutagenized T. gondii strain was divided into 3 independent cultures that were exposed to 250 or 500 nM BKI-1748 for 60 days. Clonal strains of T. gondii were selected by limiting dilution from parasites that proliferated during BKI-1748 exposure. Six clonal strains and the respective parental strains were selected for whole genome sequencing (WGS).

Nonsynonymous single nucleotide variations (SNVs) in protein coding sequences of parental clonal strains were subtracted from the clonal strains selected by BKI-1748. SNVs in each strain were compared to identify genes containing SNVs that were common between strains (Table S1). All strains possessed a mutation in the *Tg*MAPKL1 (TGGT1_312570) gene. Three unique mutations were identified in the ATP binding pocket of the *Tg*MAPKL1 gene; Leu162Gln, Ile171Thr and Ser191Thr (Figure 1A,B). SNVs were not found in *Tg*CDPK1 by analysis of direct sequencing of *Tg*CDPK1 and WGS.

**Figure 1:**
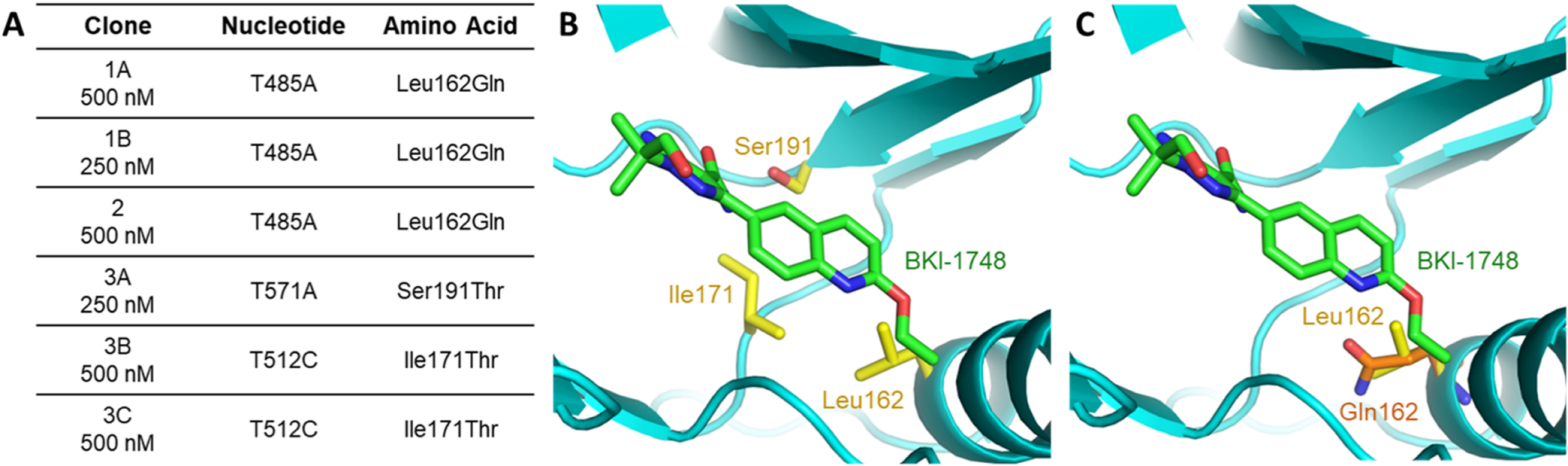
Mutations in the *Tg*MAPKL1 ATP binding site were found in all 6 BKI-1748 resistant clones. (A) Clone number and BKI-1748 selection concentration, nucleotide change, amino acid change within the *Tg*MAPKL1 (B) Prediction of the mutated amino acids (yellow) with BKI-1748 (green) in the *Tg*MAPKL1 ATP binding site. (C) Leu162Gln mutation in *Tg*MAPKL1 as visualized in PyMOL. The side-chain of Gln162 (orange) protrudes deeper into the ATP binding site than Leu162 (yellow). *Tg*MAPKL1 structure predicted by Alphafold 2.0.

To test if a SNV that was found in *Tg*MAPKL1 caused BKI-1748 resistance, the Leu162Gln substitution was introduced into the *Tg*MAPKL1 gene of an RH T. gondii strain using CRISPR-Cas9 site-directed mutagenesis and BKI-1748 at 500 nM for selection for 21 days. The clonal strain that was selected by limiting dilution was found to have 3.4-fold resistance to BKI-1748 (Table 1). The genome of this clone was sequenced and analyzed to confirm the Leu162Gln substitution, the silent nucleotide mutation in the protospacer adjacent motif (PAM) sequence and to confirm that there were no other SNVs introduced that were associated with BKI-1748 resistance. To evaluate the possibility of spontaneous mutations under selective drug pressure, T. gondii were treated with BKI-1748 at 500 nM without site-directed mutagenesis for 21 days and parasite replication was not observed.

**Table 1:**
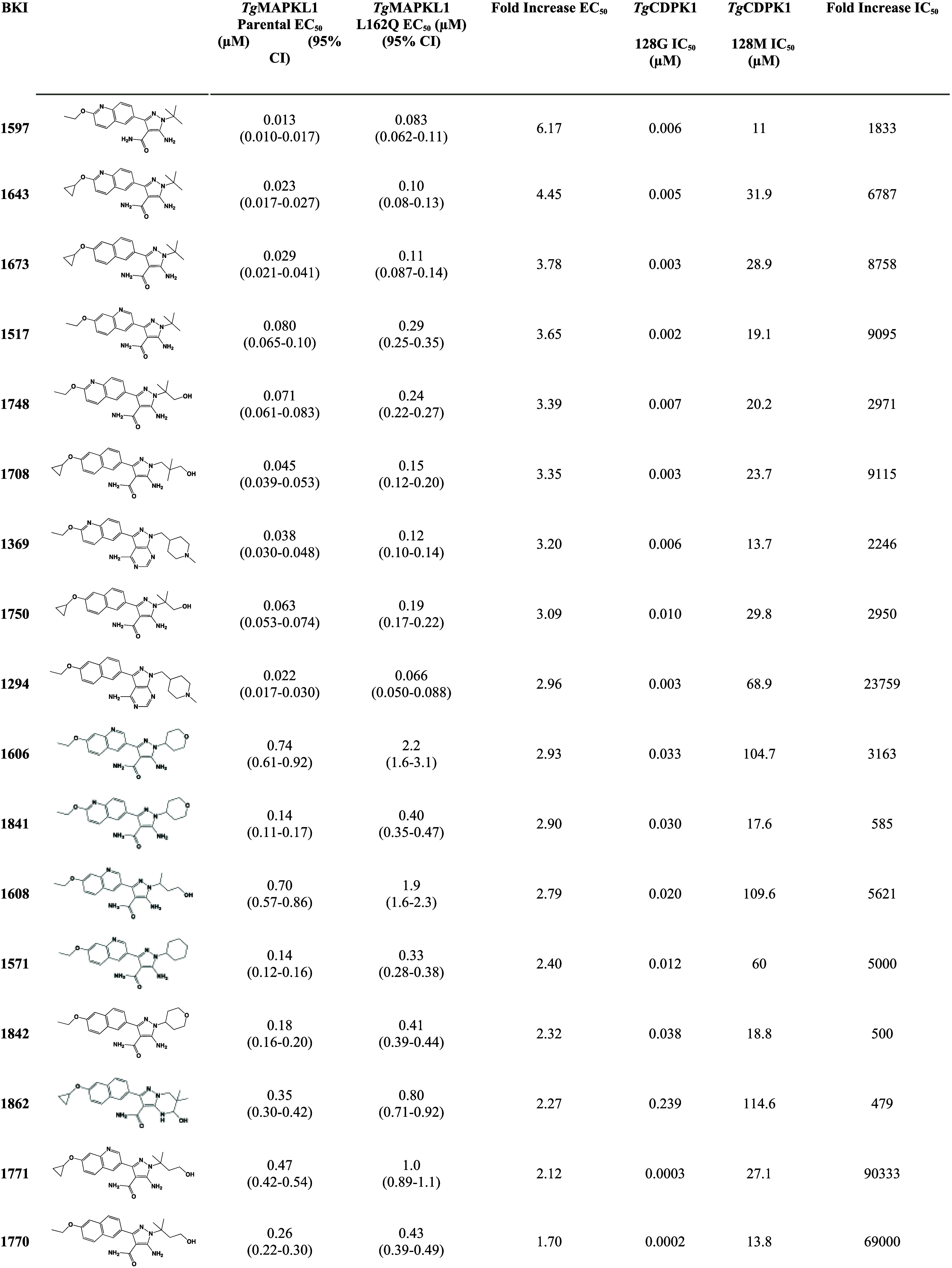
EC_50_ (50% Effective Concentration) of BKIs against T. gondii with a L162Q Substitution in *Tg*MAPKL1 compared to the Parent Strain and IC_50_ (50% Inhibitory Concentration) of *Tg*CDPK1 with a G128M Gatekeeper Residue Mutation compared to Wild Type *Tg*CDPK1^a^

a T. gondii were quantified by measurement of β-galactosidase activity. IC_50_ (50% inhibitory concentration) of BKIs against the *Tg*CDPK1 enzyme with either the wild type glycine or an introduced methionine gatekeeper residue.

### BKI Resistance Caused by the Leu162Gln Substitution in *Tg*MAPKL1

We selected a structurally varied panel of BKIs to test against the *Tg*MAPKL1^L162Q^ strain and purified *Tg*CDPK1 with and without the *Tg*CDPK1^G128M^ gatekeeper mutation that causes BKI resistance.^4^ The *Tg*MAPKL1^L162Q^ strain possessed a β-galactosidase transgene, the activity of which was measured in T. gondii inhibition assays. BKIs with pyrazolopyrimidine and 5-aminopyrazole-4-carboxamide scaffolds and varying substituents were tested. All compounds were less potent against the *Tg*MAPKL1^L162Q^ strain with nonoverlapping 95% confidence intervals compared to the parent clone. Compounds were also less potent against the purified *Tg*CDPK1^G128M^ enzyme (Table 1). A clear structure activity relationship that demonstrated compound selectivity for either target was not apparent, however, compounds that were less potent against wild type T. gondii had activity that was less diminished by *Tg*MAPKL1^L162Q^ (Figure S1).

### BKI Resistance Caused by Substitutions in *Tg*MAPKL1, *Tg*CDPK1, or Both *Tg*MAPKL1 and *Tg*CDPK1

A compound targeting multiple enzymes may maintain potency despite losing activity against one of the targets due to a resistance mutation. With this in mind, additional strains were created with amino acid substitutions in *Tg*CDPK1 and both *Tg*CDPK1 and *Tg*MAPKL1 using site-directed mutagenesis. BKIs with high and low loss of potency against *Tg*MAPKL1^L162Q^ and one of the initial *Tg*CDPK1 inhibitors, 1-NM-PP1, were tested against the single and double mutant strains (Table 2, Figure S2). The strains used for the experiments shown in Table 2 lacked β-galactosidase activity, as such, parasite proliferation was measured by counting the remaining DAPI-stained host cell nuclei. The differences in the assays used for the experiments in Tables 1 and 2 resulted in differences in EC_50_s. All compounds had modest decreases in potency against single mutations of 2 to 7-fold, however, some EC_50_ values of mutant and parental clones had overlapping 95% confidence intervals. Variability in this assay limited the definitive characterization of small changes in potency. Loss of potency was much greater against double mutants with a 9.5 to 157-fold loss of potency. The highest degree of resistance conferred by the double mutation was against 1-NM-PP1 (99-fold) and BKI-1748 (157-fold). *Tg*CDPK1 mutations caused the greatest degree of resistance to BKI-1294 and 1-NM-PP1, which both have pyrazolopyrimidine scaffolds. The loss of potency against *Tg*CDPK1 and *Tg*MAPKL1 mutations for all compounds tested was multiplied when both mutations were present. The mutations, alone or in combination, did not cause a significant decrease in parasite proliferation and host cell lysis (Figure S3).

**Table 2:** EC_50_ (50% Effective Concentration) of BKIs against Strains of T. gondii with Amino Acid Substitutions in *Tg*MAPKL1, *Tg*CDPK1, or Both Compared to the Parent Strain^a^

		***Tg*** **MAPKL1**		***Tg*** **CDPK1**		***Tg*** **CDPK1**		***Tg*** **CDPK1 G128M**		***Tg*** **CDPK1 G128M**	
	**parent**	**L162Q**		**G128M**		**G128T**		***Tg*** **MAPKL1 L162Q**		***Tg*** **MAPKL1 L162E**	
**BKI**	**EC**_**50**_**(μM)** (95% CI)	**fold increase EC**_**50**_	**EC**_**50**_**(μM)** (95% CI)	**fold increase EC**_**50**_	**EC**_**50**_**(μM)** (95% CI)	**fold increase EC**_**50**_	**EC**_**50**_**(μM)** (95% CI)	**fold increase EC**_**50**_	**EC**_**50**_**(μM)** (95% CI)	**fold increase EC**_**50**_	**EC**_**50**_**(μM)** (95% CI)
**1294**	0.23 (0.15–0.30)	2.0	0.45 (0.056–0.85)	6.5	1.5 (1.3–1.8)	7.4	1.7 (1.4–1.9)	13.9	3.2 (1.7–4.7)	16.1	3.7 (1.5–5.9)
**1597**	0.090 (0.077–0.10)	2.4	0.22 (0.086–0.35)	3.4	0.31 (0.17–0.45)	2.4	0.22 (0.048–0.39)	45.6	4.1 (2.2–5.9)	50.0	4.5 (2.0–7.0)
**1708**	0.093 (0.067–0.12)	2.3	0.21 (0.044–0.38)	2.8	0.26 (0.082–0.44)	nt	nt	30.1	2.8 (1.0–4.6)	nt	nt
**1748**	0.042 (0.030–0.055)	6.2	0.26 (0.15–0.37)	2.9	0.12 (0.097–0.15)	1.9	0.080 (0.061–0.10)	157	6.6 (3.6–9.7)	155	6.5 (3.1–9.9)
**1770**	0.33 (0.23–0.43)	2.4	0.80 (0.38–1.2)	4.2	1.4 (1.2–1.6)	3.3	1.1 (0.70–1.5)	29.4	9.7 (2.7–17)	39.4	13 (5.0–22)
**1862**	0.22 (0.10–0.33)	2.5	0.54 (0.36–0.73)	1.8	0.39 (0.32–0.46)	nt	nt	9.5	2.1 (1.7–2.5)	nt	nt
**1-NM-PP1**	0.019 (0.012–0.027)	3.9	0.075 (0.030–0.12)	7.4	0.14 (0.078–0.12)	nt	nt	89.5	1.7 (1.6–1.9)	nt	nt

a Parasite growth was quantified by measuring remaining host cell nuclei. (Mean EC_50_ with 95% confidence interval, all values derived from ≥ 3 experiments) nt: not tested.

### Comparison of *Tg*MAPKL1 and *Tg*CDPK1 Protein Structures with BKI-1748

Superposition of α Fold 2.0 model of *Tg*MAPKL1 and *Tg*CDPK1 (PDB: 4ONA) was calculated using COOT, which calculated an overall r.m.s.d. of 2.84 Ångstrom aligning 264 of 465 residues, 29.8% identity (Figure 2).^13^ Docking of BKI-1748 was conducted using GLIDE (Schrödinger LLC) using the structure of TgCDPK1 bound to BKI-1517 (PDB 4ONA) as a receptor.^14^ The structure of *Tg*MAPKL1 was predicted by AlphaFold 2.0 available from the AlphaFold Protein Structure Database through Uniprot.^15^ Top 3 GLIDE scores of docked BKI-1748 were −10.8, −10.6, and −10.3. No significant deviation of the overall core of the molecule was observed in top 5 hits. Thus, the active sites of *Tg*MAPKL1 and *Tg*CDPK1 are nearly identical and are both predicted to bind BKI-1748 similarly.

**Figure 2:**
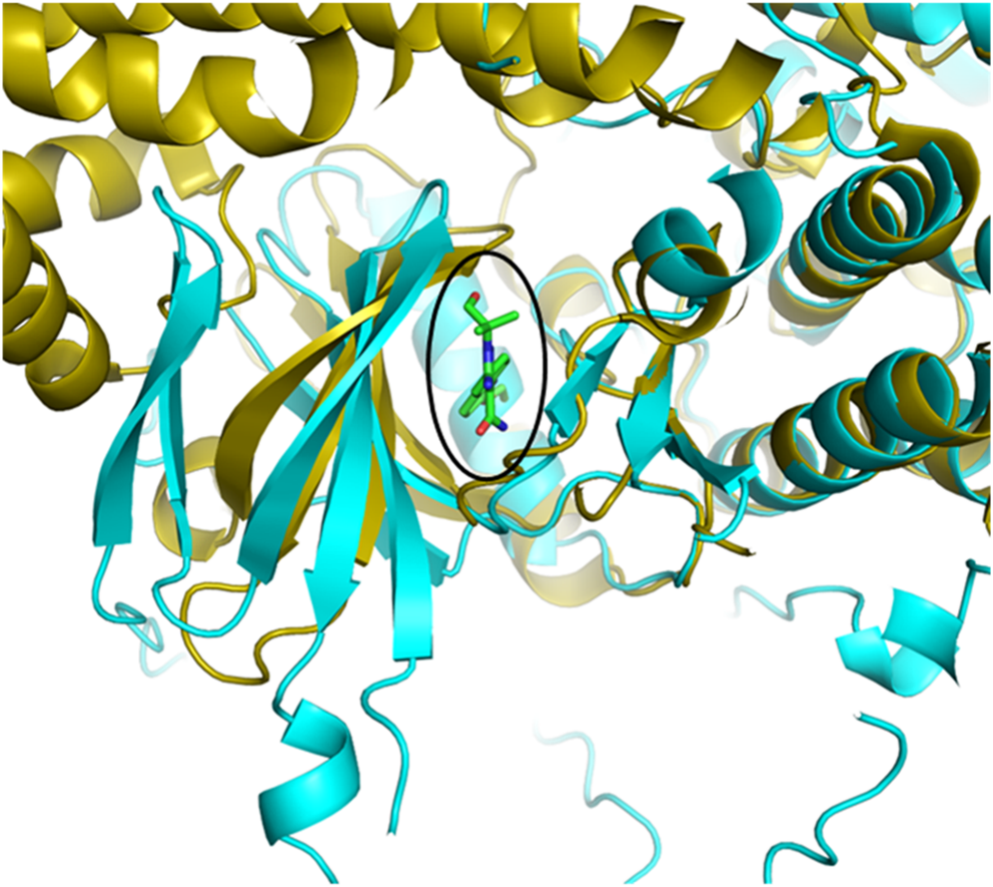
Comparison of *Tg*MAPKL1 (turquoise) and *Tg*CDPK1 (gold) with BKI-1748 (green, circled) in the ATP binding pocket. *Tg*MAPKL1 predicted by Alphafold 2.0, *Tg*CDPK1 (PDB: 4NOA).

### Comparison of Apicomplexan and Human MAPKL1 and MAPK1 Protein Structures

Apicomplexan MAPKL1/MAPK1 and human MAPK1 have substantial sequence and structural variation (Figure 3). The cyst-forming neuroinvasive apicomplexan pathogens, T. gondii, N. caninum and S. neurona have larger predicted MAPK1 sequences of 1298, 1308, and 2374 amino acids, respectively. Whereas, the sequence of C. parvum and *Homo sapiens* are smaller at 566 and 360 amino acids. *Tg*MAPKL1 shares 64% sequence identity with the entire N. caninum homologue, however, only the first 733 amino acids of *Tg*MAPKL1 align with C. parvum and share 32% identity. Similarly, the first 551 amino acids of *Tg*MAPKL1 align to the H. sapiens MAPK1 with 30% identity. T. gondii, N. caninum and S. neurona have three additional segments within the active region of the kinase that do not align with C. parvum or H. sapiens MAPK1. The highly conserved activation loop motif, TxY, is TDY in *Tg*MAPKL1 and other Apicomplexa, whereas the mammalian MAPK1 motif is TEY. The gatekeeper residue of the human MAPK1 is a larger glutamine residue compared to serine of *Tg*MAPKL1 or threonine of the putative C. parvum MAPK1.

**Figure 3:**
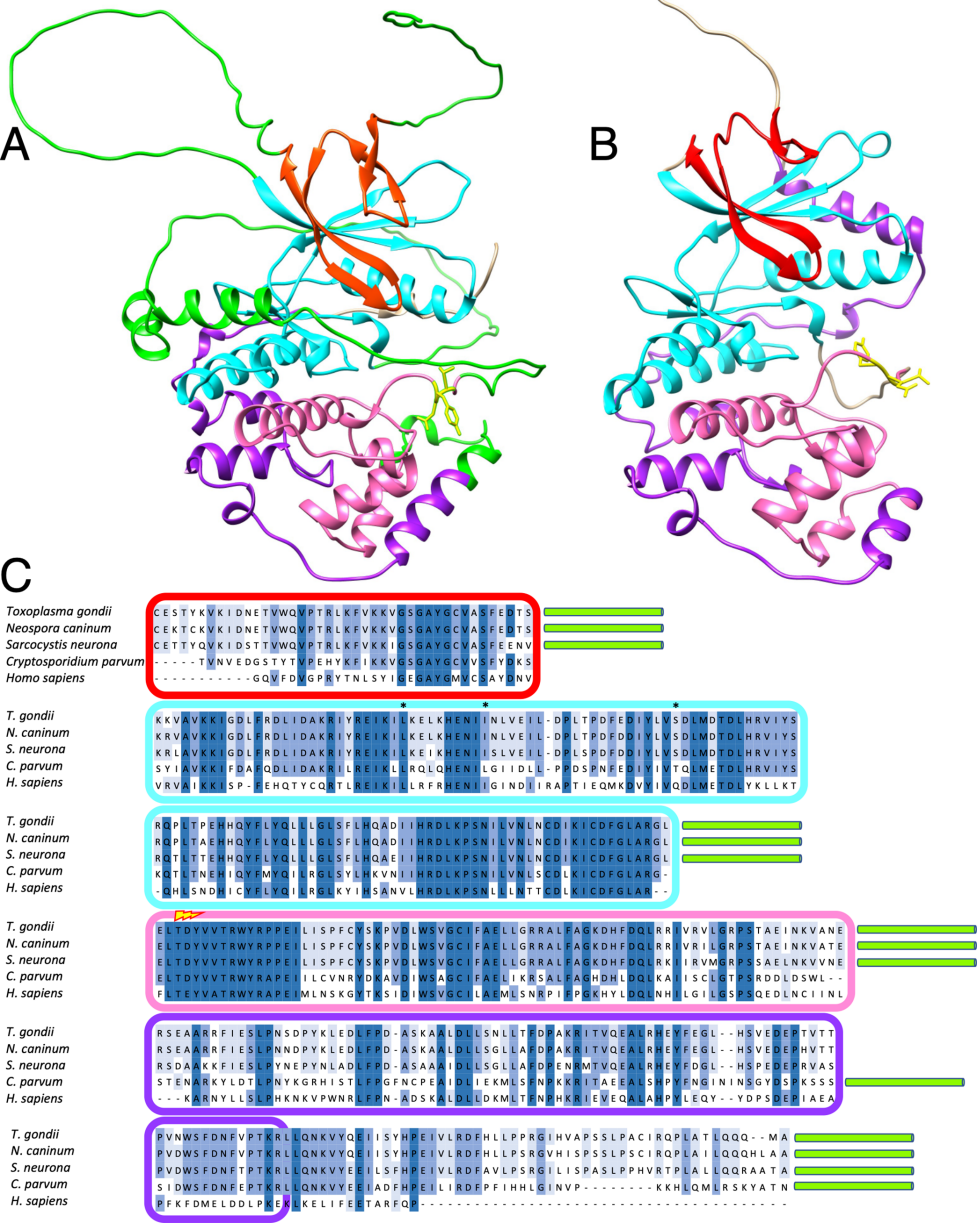
Comparison of apicomplexan MAPKL1 and human MAPK1. (A) Toxoplasma gondii MAPKL1 (AF-Q5SC61-F1-v4) α Fold predicted protein segment (532 AA). Red, Cyan, Pink and Purple: conserved sequence regions. Green regions: blocks of sequence that are not present in the human MAPK1 sequence. Yellow residues: TDY dual phosphorylation and activation site. (B) Human MAPK1 (UniProt P28482). (C) Multiple sequence alignment of human and apicomplexan MAPK1/L1. Colors correspond to protein models A, B. *, BKI resistance mutation sites; lightning bolt, phosphorylation/kinase activation site.

## Discussion

The BKI scaffold has been optimized for efficacy, pharmacokinetics, and safety to select a lead compound for toxoplasmosis. BKI-1748 has emerged as a lead preclinical compound that is safe and effective in multiple animal models of toxoplasmosis. Efficacy studies, which have included rodent and sheep models of acute, latent, and congenital toxoplasmosis, support advancement toward human use. Throughout the iterative development of hundreds of compounds, BKIs have consistently demonstrated inhibition of *Tg*CDPK1 in enzymatic assays. Lead compounds that were evaluated for off-target human kinase inhibition demonstrated a high therapeutic index between *Tg*CDPK1 and the human Src kinase, which contains a threonine gatekeeper residue.^16^ BKI compounds have also been tested in a 80-protein kinase panel representing the human protein kinome (AbbVie) and show poor activity against human protein kinases, with the exception of a small number of compounds that possessed moderate activity against protein kinase D3 (PKD3) and mitogen-activated protein kinase kinase 1 (MEK1).^17^ Of particular significance, BKI-1748, had an IC_50_ of 8.37 μM against human MAPK1. The lack of BKI-1748 activity against human MAPK1 is not surprising, given the extensive differences in gatekeeper residues and structures (Figure 3). BKIs evaluated in this study have previously been shown to lack cytotoxicity, with 50% cytotoxic concentrations (CC_50_) against HepG2 and CRL-8155 cells that were greater than 40 μM.^18–21^ BKIs were also tested against other apicomplexan CDPKs with serine and threonine gatekeepers, as well as *Tg*CDPK1^G128T^. These experiments showed that most BKIs were more potent against the wild type *Tg*CDPK1 with a glycine gatekeeper residue than the other CDPKs. The BKI mechanism of *Tg*CDPK1 inhibition was also supported by cocrystallization of *Tg*CDPK1 with BKIs.^6^ However, the potential for inhibition of multiple enzymes exists for all small molecule inhibitors, particularly when the binding site structure is conserved across different proteins.

In this work, the identification of *Tg*MAPKL1 mutations that cause resistance to BKIs through a forward genetic screen demonstrated that a structurally diverse selection of BKIs target *Tg*MAPKL1. *Tg*MAPKL1 has a serine gatekeeper residue making it a prime target for BKIs.^16,22^ This mechanism is further supported by the multinucleated morphology of BKI-treated T. gondii and N. caninum and the reduction of brain tissue cysts in a mouse model of latent infection.^11,23^ Inhibiting the defined functions of *Tg*CDPK1 (microneme secretion, motility, invasion, and egress from host cells) would not necessarily be expected to cause defects in replication.^6^ However, *Tg*MAPKL1 is necessary for completion of the T. gondii cell cycle. Temperature sensitive *Tg*MAPKL1 mutants arrested growth at 40 °C and demonstrated defects in the coordination of daughter budding with mitosis leading to abnormal numbers of internal daughters and nuclei.^24^ This morphology resembled BKI-treated parasites.^11^ Moreover, a *Tg*MAPKL1 inhibitor, SB505124, also arrested the T. gondii cell cycle.^25^ Additionally, a previous *Tg*MAPKL1 mutation was selected through a forward genetic screen, and the introduction of an S191Y substitution conferred resistance to the BKI, 1-NM-PP1.^26^

Further validation of *Tg*CDPK1 and *Tg*MAPKL1 as drug targets comes from gene disruption. A whole genome gene deletion screen found that both genes were fitness conferring, with *Tg*MAPKL1 and *Tg*CDPK1 phenotype scores of −5.5 and −3.3, respectively, on a scale of 2.96 to −6.89, with the more negative number indicating a greater role in fitness.^27^ In single gene knock out experiments, disruption of *Tg*MAPKL1 led to 44% fewer parasites at 96 h and smaller parasitophorous vacuoles.^28^ Similarly, a conditional knockdown of *Tg*CDPK1 demonstrated that it was essential for the T. gondii lytic cycle.^6^ Altogether, the results we report, combined with prior studies, suggest that *Tg*CDPK1 and *Tg*MAPKL1 are validated drug targets and that dual inhibition of *Tg*MAPKL1 and *Tg*CDPK1 is common for BKIs.

The TgMAPKL1^L162Q^ mutation did not cause a gross phenotypic effect when parasites were cultured without inhibitors. We did not observe an altered morphology or reduced proliferation in the mutant strains under standard culture conditions, however, it is possible that these mutations would have an effect in vivo or in nutrient-limited environments. Given that many protein kinases have larger gatekeeper residues, the introduction of the *Tg*CDPK1^G128M^ mutation may not affect the protein kinase’s ability to bind ATP. Similarly, the *Tg*MAPKL1^L162Q^ mutation in the ATP binding pocket may disrupt BKI binding but have little to no impact on ATP. Modeling of BKI-1748 in the MAPKL1 pocket (Figure 1) suggests that the more rigid bicyclic ring structure of BKI-1748 clashes with the glutamine at position 162, whereas, the more flexible triphosphate portion of ATP may not be hindered. Importantly, the forward genetic screen that selected for BKI-1748 resistance mutations would be expected to select for mutations that do not limit T. gondii proliferation and parasites with these mutations would out-compete parasites with mutations that slow proliferation.

Unlike previous compounds that have been shown to inhibit *Tg*MAPKL1, the BKIs we evaluated are highly potent and effective in vivo, confirming that *Tg*MAPKL1 is a promising target for therapeutic development. While MAPKs are found across eukaryotes, apicomplexan MAPKs are distinct from MAPKs found in other eukaryotes.^29^ MAPKL1s in T. gondii and closely related apicomplexan pathogens are structurally divergent from mammalian MAPKs, with large C-terminal extension and additional segments in the activation loop region of the protein (Figure 3).^29^ The N-terminal 533 amino acid segment of *Tg*MAPKL1 that contains the catalytic core has been shown to be kinetically active without the C-terminal half, however, epitope tagging of the C-terminal end of *Tg*MAPKL1 and immunofluorescence microscopy has demonstrated localization of *Tg*MAPKL1 to the centrosomal region of dividing parasites.^24,30^ Additionally, differences are found in the highly conserved activation loop motif, TxY, the gatekeeper residue, overall protein size, and mechanism of phosphorylation and function compared to human MAPK1. Significant structural differences between apicomplexan MAPKL1s and mammalian MAPKs reveal unique apicomplexan elements that may be exploited by parasite-selective BKIs.

Three MAPK orthologs have been identified in T. gondii: MAPKL1, ERK7 (TGME49_233010) and MAPK2 (TGME49_207820.) Both ERK7 and MAPK2 are potential drug targets based on experiments that have shown that they are important for parasite survival.^31,32^ Alignment of the three T. gondii MAPK orthologs suggests that MAPK2 has a leucine gatekeeper residue and ERK7 has a phenylalanine gatekeeper residue. These larger gatekeeper residues may prevent BKIs from binding in the ATP active site. Depletion of MAPK2 has been shown to arrest replication before centrosome duplication and daughter cell budding, and depletion of ERK7 causes major cytoskeletal defects at the apical pole.^31,32^ Both of these phenotypes are distinct from the multinucleated phenotype of MAPKL1 depletion and BKI inhibition.^9,11^ Whereas BKI inhibition of MAPK2 or ERK7 cannot entirely be excluded, the high level of resistance in the double MAPKL1/CDPK1 mutants, the difference in MAPK gatekeeper residue sizes, and the parasite morphology that has previously been observed with BKI inhibition indicate that MAPKL1 and CDPK1 are the primary targets of BKIs evaluated in this study against T. gondii.

BKIs have also been highly effective against pathogenic Cryptosporidium species.^33^ C. parvum has multiple MAPK orthologs, with the gene, cgd3_3030, being the most similar to *Tg*MAPKL1. The gatekeeper residue of cgd3_3030 is a threonine, a small residue, like the serine gatekeeper residue in *Tg*MAPKL1. However, the BKI-1748 resistance caused by the *Tg*MAPKL1 S191T mutation found in our mutagenesis screen suggests that some BKIs may be inherently less active against C. parvum than T. gondii due to the larger threonine gatekeeper residue found in C. parvum cgd3_3030. The Plasmodium falciparum MAPK1 has a bulky phenylalanine gatekeeper residue, which would limit BKI inhibition. Additionally, *Pf*MAPK homologues have been shown to be nonessential for schizogony.^34^ Unlike T. gondii, the putative C. parvum MAPK ortholog only has one prominent unique extension and is predicted to be 566 amino acids, half of the size of the *Tg*MAPKL1. The possibility that BKIs also inhibit this putative Cryptosporidium MAPK requires further investigation.

Structural optimization and determination of target specificity for compounds with multiple targets is challenging. Assays that test compounds against isolated target enzymes may not accurately predict compound activity in the cell and assay conditions may not replicate the composition of the target’s intracellular compartment. Analysis of resistance-conferring mutations can be a valuable tool to identify proteins that interact with an inhibitor. However, resistance mutations may occur in proteins that are not targets, but instead only cause resistance, such as efflux transporters like *Pf*CRT (Plasmodium falciparum chloroquine resistance transporter). Both *Tg*MAPKL1 and *Tg*CDPK1 are likely BKI targets rather than solely mediators of resistance given their functions within T. gondii and the resulting phenotype from BKI exposure. However, analysis of drug resistance mutations to determine target selectivity is limited by multiple factors. Foremost, each compound possesses a different affinity for each target, and each resistance mutation confers varying levels of resistance to individual compounds.

BKI inhibition of both *Tg*CDPK1 and *Tg*MAPKL1 illustrates how a compound may retain activity against a parasite when it loses activity against one target but remains active against the second target. In this case, the loss of activity against *Tg*CDPK1 or *Tg*MAPKL1 is masked because activity against the other target is sufficient to inhibit proliferation. The leap in resistance from 3- or 6-fold to more than 150-fold when there are resistance mutations in both sites demonstrates that resistance to BKI binding at a particular target is greater than what is observed with single target mutations. The development of resistance mutations under selective drug pressure with or without mutagenesis may point to a primary target. In our screen, as well as prior screens of 1-NM-PP1 and other inhibitors, *Tg*MAPKL1 mutations emerged instead of *Tg*CDPK1. This finding could suggest that *Tg*MAPKL1 is a more consequential target for BKIs wherein parasites must acquire a resistance mutation at this site to survive. However, the recovery of *Tg*MAPKL1 mutants could also be the result of greater parasite tolerance for mutations at the *Tg*MAPKL1 ATP binding site, compared with the *Tg*CDPK1 ATP binding site or the compound concentration used for selection. In this case, the creation of a strain with mutations in both active sites was key to understanding the importance of each target.

The BKI series of compounds, particularly BKI-1748, are promising late-stage preclinical compounds for toxoplasmosis and cryptosporidiosis. Fully understanding the mechanisms of antiparasitic compounds is essential in optimizing efficacy, anticipating toxicity, and identifying combination therapies. Our results show that *Tg*MAPKL1 and *Tg*CDPK1 inhibition is a prominent mechanism of all BKIs tested, suggesting that selective inhibition of the ATP-binding site of only one of these two targets may be difficult to attain. Notably, these studies raise the possibility that the majority of BKIs may inhibit both *Tg*CDPK1 and *Tg*MAPKL1. The advantage of targeting two enzymes is potentially a higher genetic barrier to resistance than a single target. The disadvantage of antiparasitic compounds that have multiple targets is toxicity related to inhibition of homologous host targets or off-target inhibition. Concerning BKIs, *Tg*CDPK1 does not have a human homologue and *Tg*MAPKL1 has significant structural differences from human MAPK1. BKI-1748 has undergone extensive in vitro and in vivo toxicity testing that has not found significant toxicity or human MAPK1 inhibition. Discovering that BKIs are dual kinase inhibitors is a vital component of advancing these promising compounds toward use for human and veterinary parasitic infections.

## Methods

### Host Cell and T. gondii Culture

Human Foreskin Fibroblasts (Primary Dermal Fibroblast Normal; Human, Neonatal) were acquired from ATCC (PCS-201–010). Mycoplasma contamination certification and cell line authentication per ATCC. Human Foreskin Fibroblasts (HFF) were cultured in Dulbecco Modified Eagle Media (DMEM), with d-Glucose (4.5 g/L), l-glutamine, and sodium pyruvate (110 mg/L) (Gibco) that were further supplemented with 10% Fetal Clone 1 serum (FCS)­(HyClone), 50 U/mL penicillin (Gibco), 50 μL/mL streptomycin (Gibco), and 1x GlutaMax (Gibco). HFF cells were cultured in T25 flasks (Corning) in a humidified incubator at 37 °C and 5% CO_2_. For T. gondii culture, media was replaced with DMEM, as described above, with 1% FCS.

### T. gondii Proliferation Inhibition Assays of BKIs against the *Tg*MAPKL1^L162Q^ Strain (Table 1)

For proliferation inhibition assays, a RH T. gondii strain expressing a β-galactosidase transgene was used for CRISPR/Cas9 site directed introduction of the L162Q substitution. The parental strain and the mutant strain were tested simultaneously. Compounds were dissolved in DMSO and serially diluted 4-fold across the first 11 wells of a 96 well plate, with the final drug concentration in the first column being 25 μM. The last column was left as a no drug control. T. gondii was then added to the 96 well plate at a density of 4000 parasites per well. Plates were incubated 72 h at 37 °C and 5% CO_2_. T. gondii were quantified spectrophotometrically by measuring the β-galactosidase concentration of each well after cell lysis and exposure to chlorophenol red β-d-galactopyranose (Sigma-Aldrich, St. Louis, MO). Each compound was tested at least three times in quadruplicate.

### T. gondii CDPK1 Enzyme Inhibition Assays (Table 1)

Expression, purification, and enzymatic evaluation of wild type and Gly128Met gatekeeper mutant *Tg*CDPK1 was performed as described previously.^4^ Briefly, enzymatic reactions were performed with 4 nM of either wild type or Gly128Met *Tg*CDPK1 in assay buffer containing 20 mM HEPES (pH = 7.5), 0.1% BSA, 10 mM MgCl2, 1 mM EGTA, 2 mM CaCl_2_, 10 μM ATP, and 40 μM Syntide-2 peptide substrate. After 90 min at 30 °C, the enzymatic reactions were terminated by adding EGTA to a final concentration of 5 mM. The amount of ATP remaining in solution was evaluated using the Kinase Glo luciferase assay (Promega, Madison, WI), with sample luminescence read using a Microbeta 2 plate reader (PerkinElmer, Waltham, MA). Results were converted to percent inhibition and IC_50_ values were calculated using nonlinear regression analysis in GraphPad Prism. Compounds were evaluated in triplicate in 8-point dilutions (3-fold dilution series) during the enzymatic reactions.

### T. gondii Proliferation Inhibition Assays of BKIs Comparing *Tg*MAPKL1, *Tg*CDPK1, and Combined Mutations (Table 2, Figure S2)

Compounds were dissolved in DMSO and serially diluted either 4-fold or 2-fold across the first 10 wells of a 96 well plate, with the final drug concentration in the first column being 25 μM. The strain of T. gondii was then added to the first 11 columns of the 96 well plate at a density of 4000 parasites per well. The 11th column was left as a no drug control and the 12th column was left as a no drug and no parasite control. After 5 days of incubation at 37 °C and 5% CO_2_, cells were washed with phosphate-buffered saline (PBS) and fixed with 4% PFA in PBS (Thermo Fisher Scientific, Waltham, MA). After fixing, cells were washed three times with PBS, and stained with 300 nM DAPI in PBS before being washed again three times with PBS.

Images were acquired in an ImageXpress Pico Automated Cell Imaging System (Molecular Devices, San Jose, CA) with a 4× objective using UV fluorescence. Following imaging, ImageXpress software was used to count objects with a fluorescence intensity ≥8 and a size of 12–30 μm, giving a total cell count for each well. The EC_50_ was determined based on cell lysis using a nonlinear regression analysis with GraphPad Prism software version 10.0.0 for Windows (GraphPad Software, Boston, MA). Each compound was tested ≥3 experiments in quadruplicate rows.

### Chemical Mutagenesis and Selection

The concentration of N-ethyl-N-nitrosourea (ENU, MilliporeSigma) required to kill 70% of a population of RH-strain T. gondii tachyzoites stably expressing β-galactosidase and green fluorescent protein (gift of Vern Carruthers, University of Michigan) was determined and found to be 2 mM. A clonal strain of these tachyzoites was isolated by limiting dilution and is hereafter referred to as the parental strain.

Approximately 1e7 tachyzoites were exposed to 2 mM ENU for 4 h in DMEM containing GlutaMAX and penicillin-streptomycin (Gibco) diluted per the manufacturer’s instructions. Tachyzoites were washed three times with PBS and transferred to a T25 flask containing DMEM, GlutaMAX, penicillin-streptomycin, and 1% FetalClone serum (Cytiva Life Sciences) in which human foreskins fibroblasts (HFFs) had previously been grown to confluence.

After 24 h of ENU exposure, BKI-1748 was added to 500 nM concentration and the flask observed for signs of parasite growth and plaque formation. When plaques became visible, the supernatant was removed and serially passaged through two more T25s prior to the isolation of clones by limiting dilution in a 96-well plate. BKI-1748 was present at 500 nM until clones were recovered. A control T25 of tachyzoites not treated with ENU failed to show any signs of growth over this time. The EC_50_ of the recovered clones was tested against BKI-1748 and compared to that of the parental strain using Student’s *t* test to verify resistance.

### Whole-Genome Sequencing and Analysis

Genomic DNA was isolated from resistant clones and the parental strain (Qiagen DNeasy Blood and Tissue Kit) following the manufacturer’s protocol and sequenced (paired-end 150 bp reads, Illumina NovaSeq 6000, Novogene Inc.) to provide at least 15x read depth. Trimmed sequences were aligned with bowtie2 ^35^ to the T. gondii GT1 genome, duplicates marked with Picard (Picard toolkit, Broad Institute), and variants called against the *Tg*GT1 genome with freebayes (arXiv:1207.3907). Variants were annotated with snpEff^36^ and filtered to a quality of >20 with vcftools.^37^ Parental strain single-nucleotide variants (SNVs) were subtracted from those in each resistant clonal strain. Genes containing SNVs common to all resistant mutants sorted by phenotypic scores.^27^ All genes containing nonsynonymous SNVs identified in resistant clones are listed in Table S1. A Venn diagram of genes containing nonsynonymous SNVs was created to show gene overlap with the Venn diagram tool https://bioinformatics.psb.ugent.be/webtools/Venn/ in figure S4.

### CRISPR/cas9 Mutagenesis and Selection

CRISPR guide RNAs were designed with the Eukaryotic Pathogen CRISPR gRNA Design Tool^38^ and introduced into pSAG1::CAS9-U6::sgUPRT (gift of David Sibley, addgene #54467) using a Q5 site-directed mutagenesis kit (New England Biolabs, Ipswich, MA). Alt-R HDR donor single strand DNA oligomers (IDT, Coralville, IA) were designed to introduce mutations of interest into MAPKL1 and CDPK1 while silently mutating the protospacer adjacent motif (PAM) (see Table S1). 40 μg of the relevant plasmid and 100 μg of the relevant donor oligomer were electroporated into RH-strain T. gondii tachyzoites (BTX Electro Cell Manipulator, 2.5 kV/resistance setting, 24 Ω, 2.0 kV)^39^ and the transformed parasites allowed to recover overnight in a T25 in which HFFs had previously been grown. The following day, BKI-1748 was added to 500 nM concentration. The CDPK1/MAPKL1 double mutant was created by introducing the CDPK1 Gly128Met mutation and selecting with 100 nM BKI-1748 followed by isolation of a clonal strain by limiting dilution. The presence of the *Tg*CDPK1 Gly128Met mutation was confirmed by direct sequencing. The *Tg*MAPKL1 Leu162Gln mutation was then introduced, as described above, and was selected for with 500 nM BKI-1748. Attempts were made to introduce the *Tg*MAPKL1 Leu162Gln first, followed by the *Tg*CDPK1 Gly128Met mutation, however, T. gondii did not survive selection.

### Protein Modeling and Alignment

PyMOL (Schrödinger LLC) software was used for structure visualization and comparison. Docking of BKI-1748 was conducted using GLIDE (Schrödinger LLC) using the structure of *Tg*CDPK1 bound to BKI-1517 (PDB 4ONA) as a receptor.^14^ The structure of *Tg*MAPKL1 was predicted by AlphaFold 2.0, available from the AlphaFold Protein Structure Database through Uniprot.^15^ Superposition of AF2 model of *Tg*MAPKL1 and *Tg*CDPK1 (PDB: 4ONA) was calculated using COOT.^13^ Alignment of T. gondii (TGGT1_312570), N. caninum (NCLIV_056080), S. neurona (SN3_00202170), C. parvum (cgd3_3030) and H. sapiens (NP_002736.3) protein sequences were aligned using MUSCLE.^40^ For Figure 3, the predicted *Tg*MAPKL1 protein structure (Q5SC61) and H. sapiens MAPK1 (P28482) were obtained from the AlphaFold Protein Structure Database and molecular graphics were performed with UCSF Chimera (University of California, San Francisco).^41^

## Supplementary Material



## Notes

Graphical comparison of fold change resistance of *Tg*MAPKL1^L162Q^ compared to EC_50_ of the parental strain; dose–response curves for EC_50_s found in Table 2; relative growth rates of mutant strains created in this manuscript, SNVs identified during chemical mutagenesis, and oligonucleotides used (PDF)
