# Supplementary material for: Bumped Kinase Inhibitors Inhibit both Toxoplasma gondii MAPKL1 and CDPK1
Source: ACS Infect Dis. 2025 May 23;11(6):1552–62. doi: 10.1021/acsinfecdis.5c00051 (PMC12172689; doi:10.1021/acsinfecdis.5c00051)
Supplement: Supplementary file 1 [file id5c00051_si_001.pdf]

Supporting Information

Bumped Kinase Inhibitors Inhibit both *Toxoplasma gondii* MAPKL1 and CDPK1

Jemma A Montgomery[1], P. Holland Alday[1,2], Ryan Choi[3], Monique Khim[4,5], Bart L. Staker[4,5], Matthew A. Hulverson[3], Kayode K. Ojo[3], Erkang Fan[6], Wesley C. Van Voorhis[3], J. Stone Doggett[1,2]*

[1] *Division of Infectious Diseases, Oregon Health and Sciences University, Portland, Oregon, 97239, USA*

[2] *Division of Infectious Diseases, Portland VA Medical Center, Portland, Oregon, 97239, USA*

[3] Center for Emerging and Re-emerging Infectious Diseases (CERID), Division of Allergy and Infectious Diseases, Department of Medicine, University of Washington, Seattle, WA, 98109, USA.

[4]Seattle Structural Genomics Center for Infectious Disease (SSGCID), Seattle, Washington, 98109, USA.

[5]Center for Global Infectious Disease Research, Seattle Children's Research Institute, Seattle, Washington, 98109, USA.

[6] Department of Biochemistry, University of Washington, Seattle, Washington, 98109, USA.

*Corresponding author: doggettj@ohsu.edu

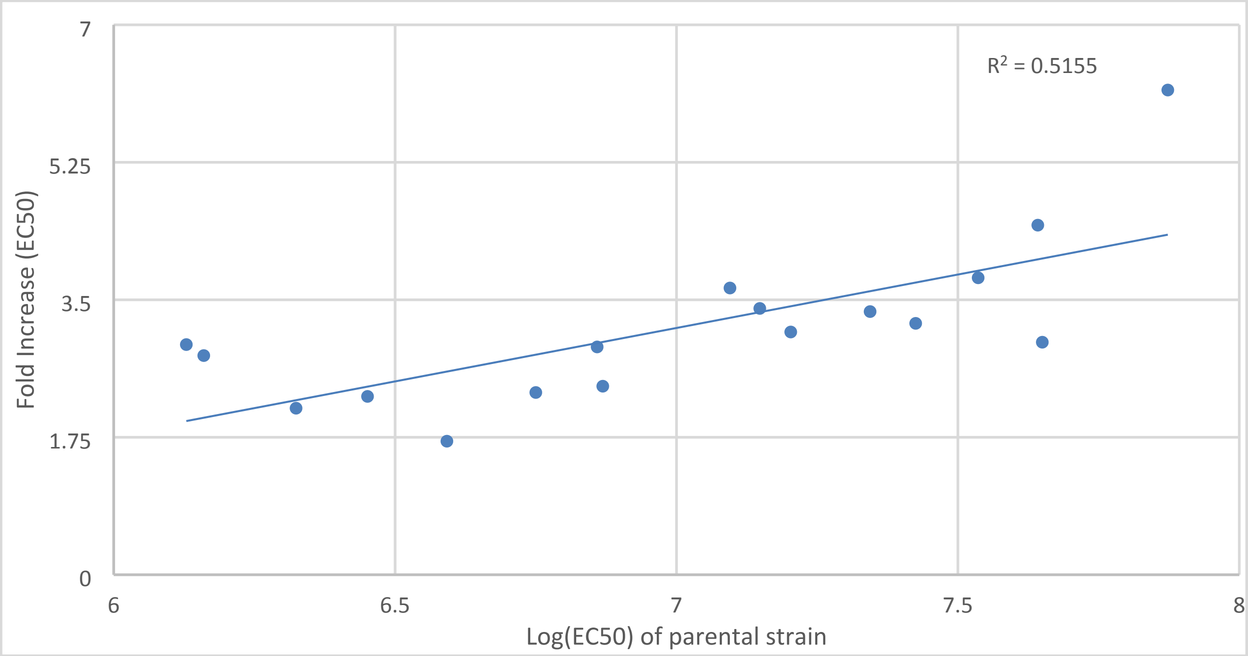

Figure S1: Fold increase EC_{50} (50% effective concentration) of TgMAPKL1 L162Q strain compared to its parental strain plotted as a function of the log(EC_{50}) of the parental strain. R^2 value of the linear regression plotted in righthand corner, statistical analysis performed using Microsoft Excel 16.89.1.

BKI 1294

MAPKL1 L162Q

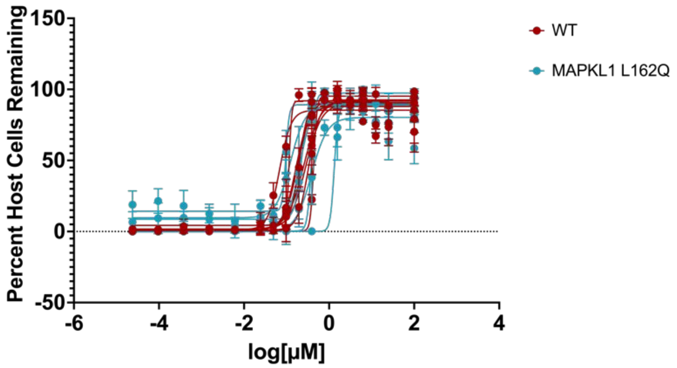

CDPK1 G128M

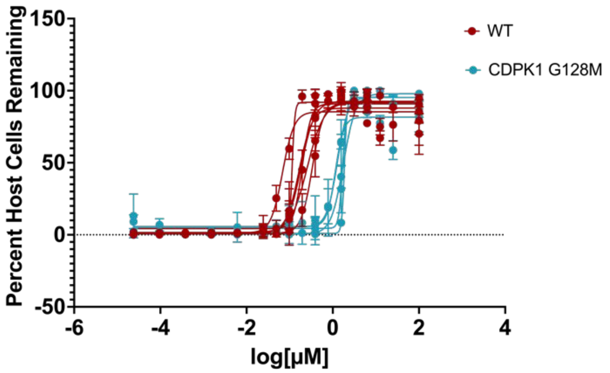

CDPK1 G128T

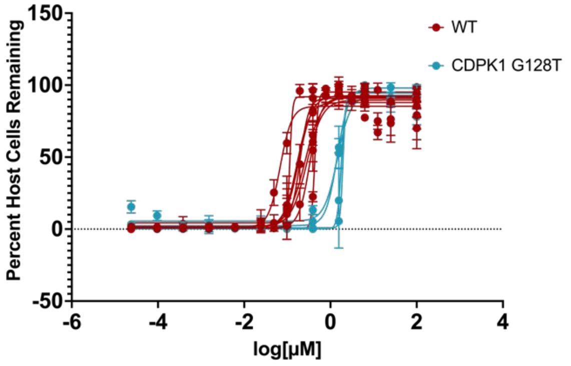

CDPK1 G128M
MAPKL1 L162Q

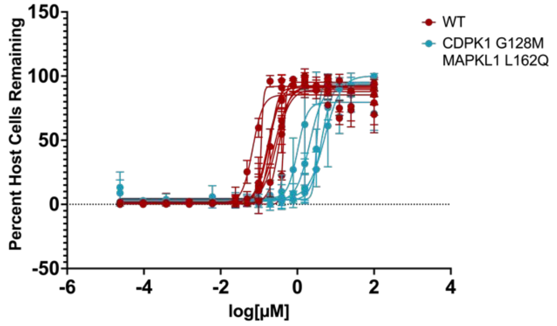

CDPK1 G128M
MAPKL1 L162E

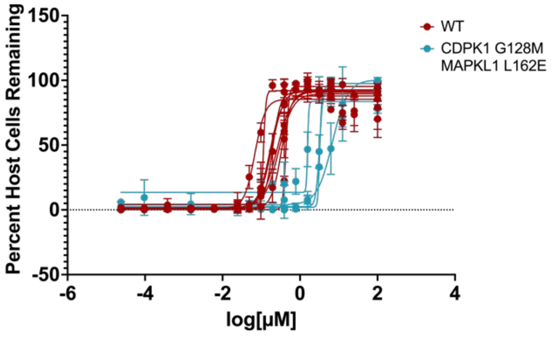

BKI 1597

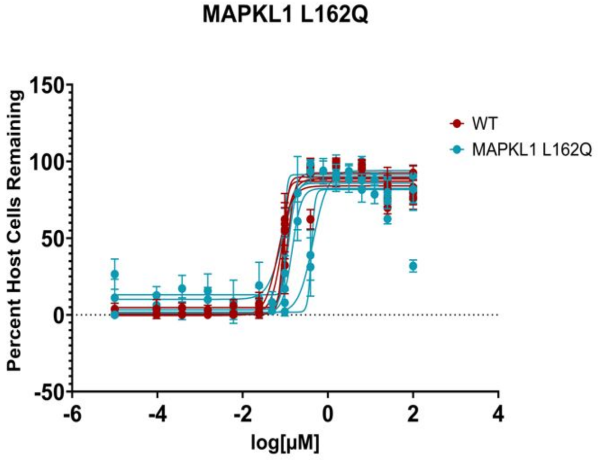

MAPKL1 L162Q
Percent Host Cells Remaining
log[µM]
WT
MAPKL1 L162Q

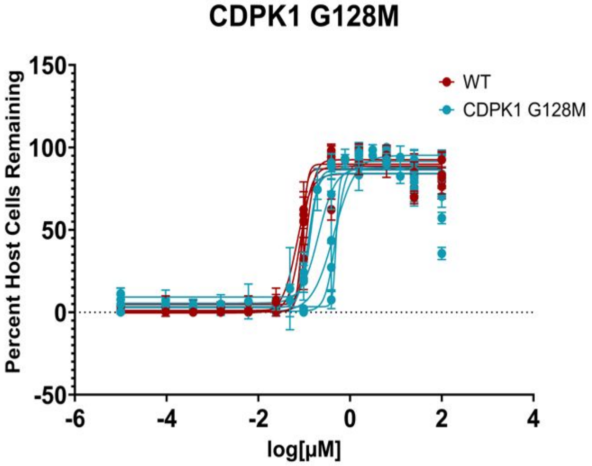

CDPK1 G128M
Percent Host Cells Remaining
log[µM]
WT
CDPK1 G128M

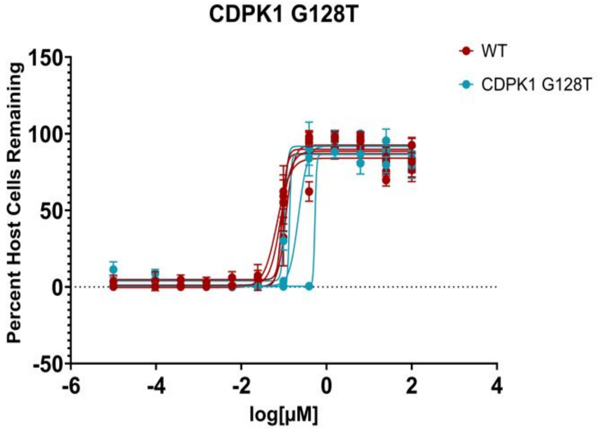

CDPK1 G128T
Percent Host Cells Remaining
log[µM]
WT
CDPK1 G128T

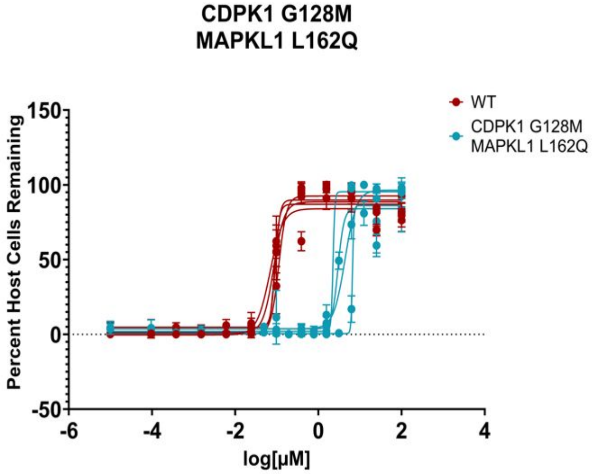

CDPK1 G128M
MAPKL1 L162Q
Percent Host Cells Remaining
log[µM]
WT
CDPK1 G128M
MAPKL1 L162Q

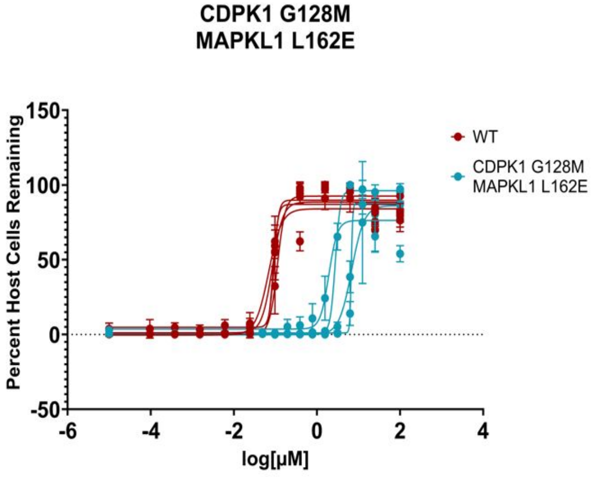

CDPK1 G128M
MAPKL1 L162E
Percent Host Cells Remaining
log[µM]
WT
CDPK1 G128M
MAPKL1 L162E

BKI-1708

MAPKL1 L162Q

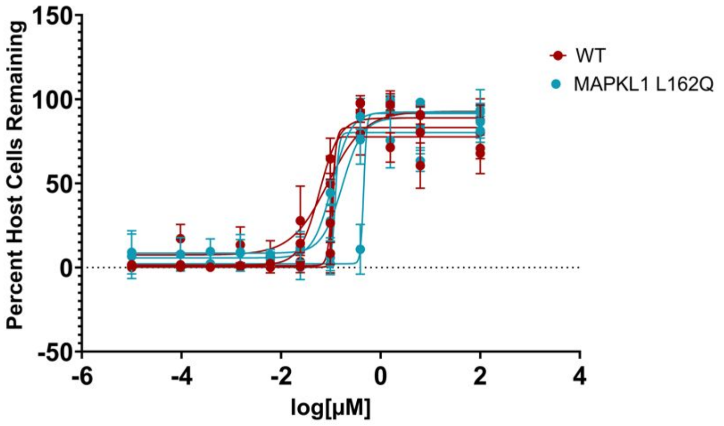

CDPK1 G128M

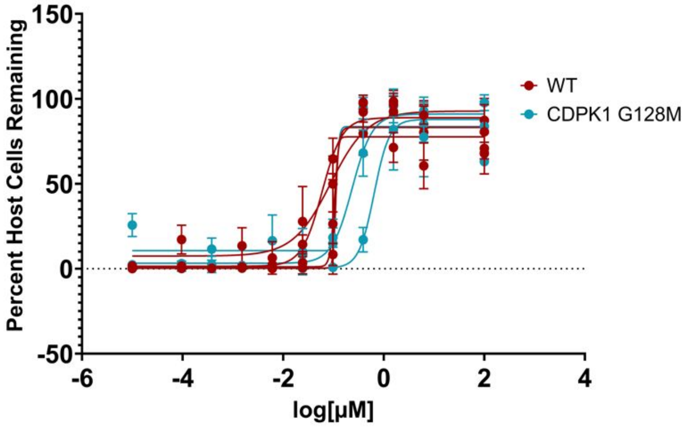

CDPK1 G128M
MAPKL1 L162Q

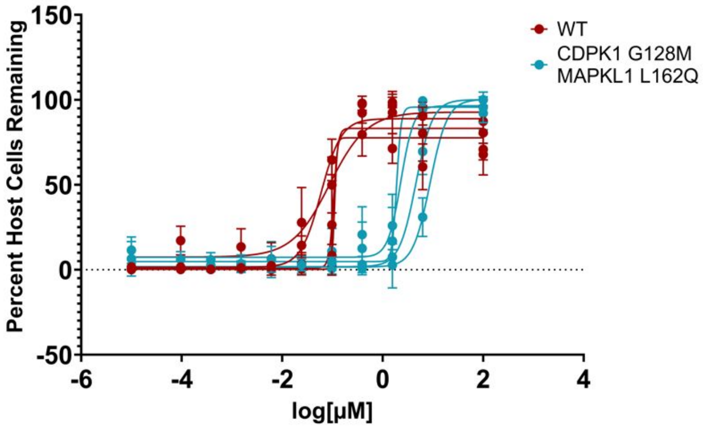

BKI 1748

MAPKL1 L162Q

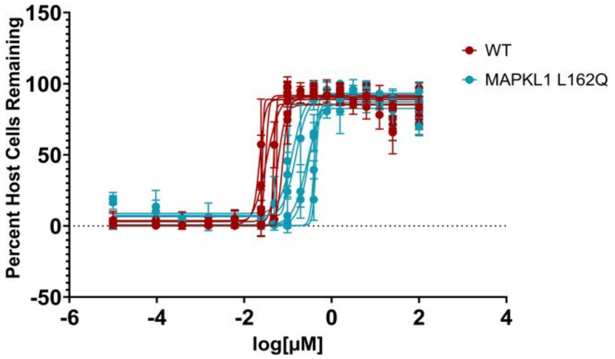

CDPK1 G128M

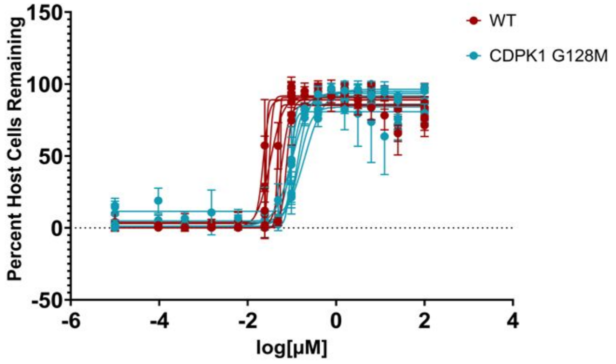

CDPK1 G128T

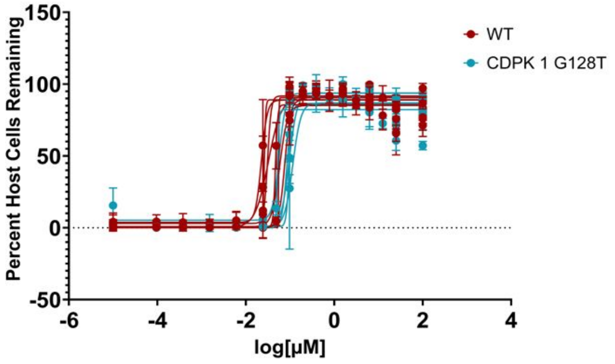

CDPK1 G128M
MAPKL1 L162Q

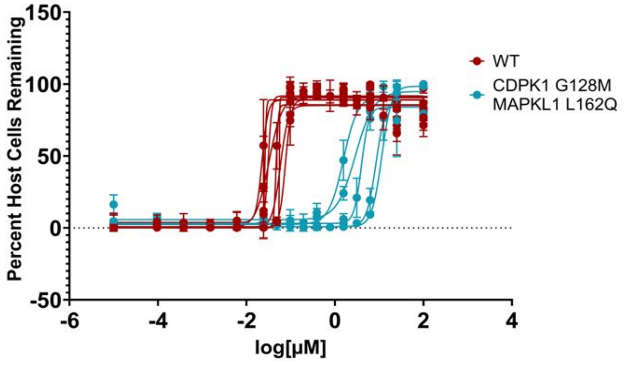

CDPK1 G128M
MAPKL1 L162E

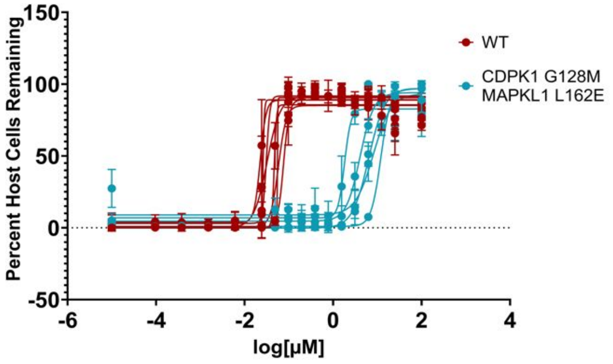

BKI 1770

MAPKL1 L162Q

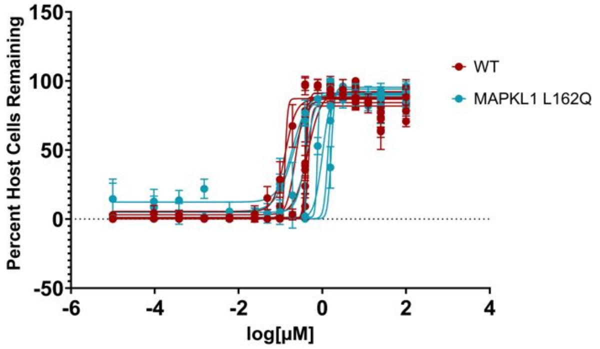

CDPK1 G128M

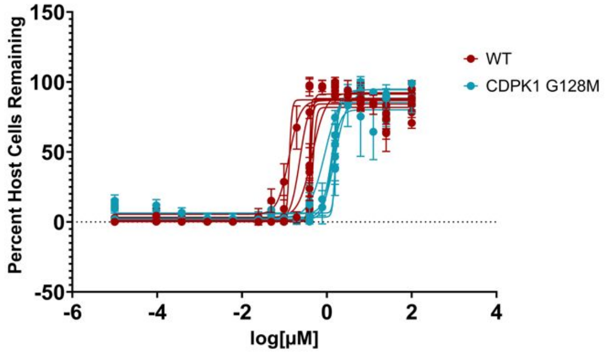

CDPK1 G128T

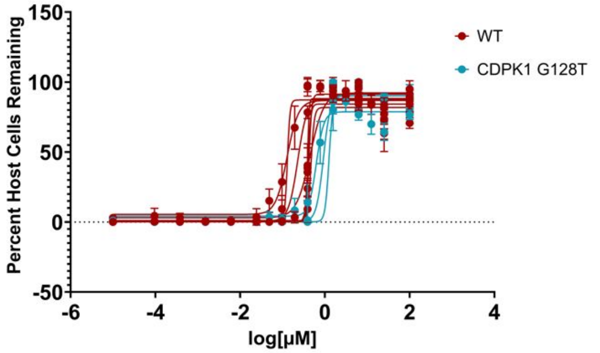

CDPK1 G128M MAPKL1 L162Q

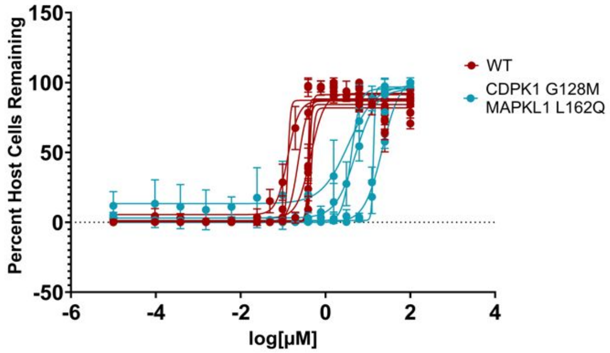

CDPK1 G128M MAPKL1 L162E

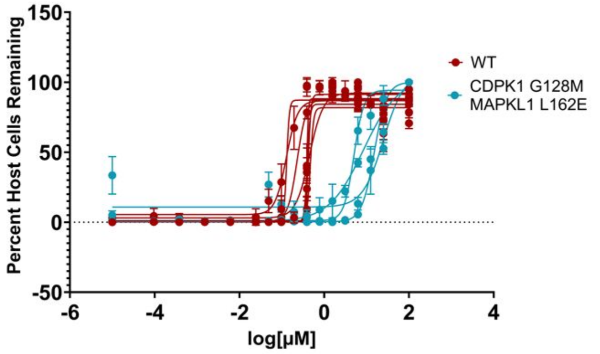

BKI-1862

MAPKL1 L162Q

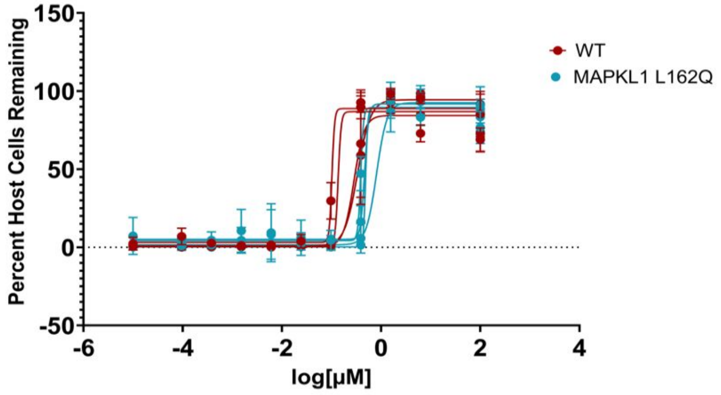

CDPK1 G128M

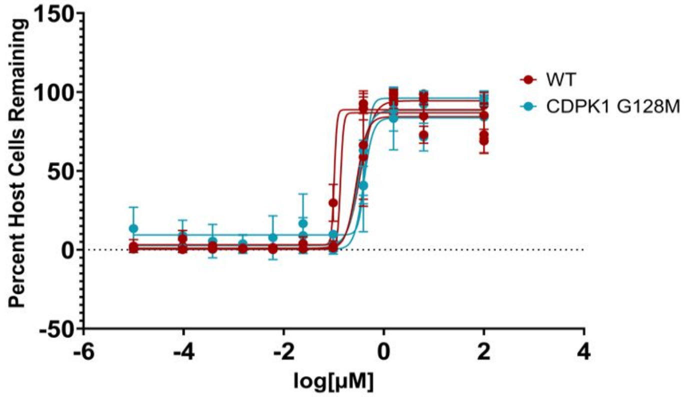

CDPK1 G128M
MAPKL1 L162Q

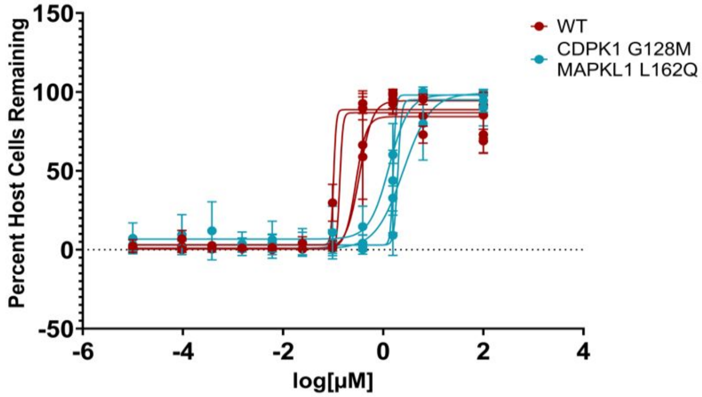

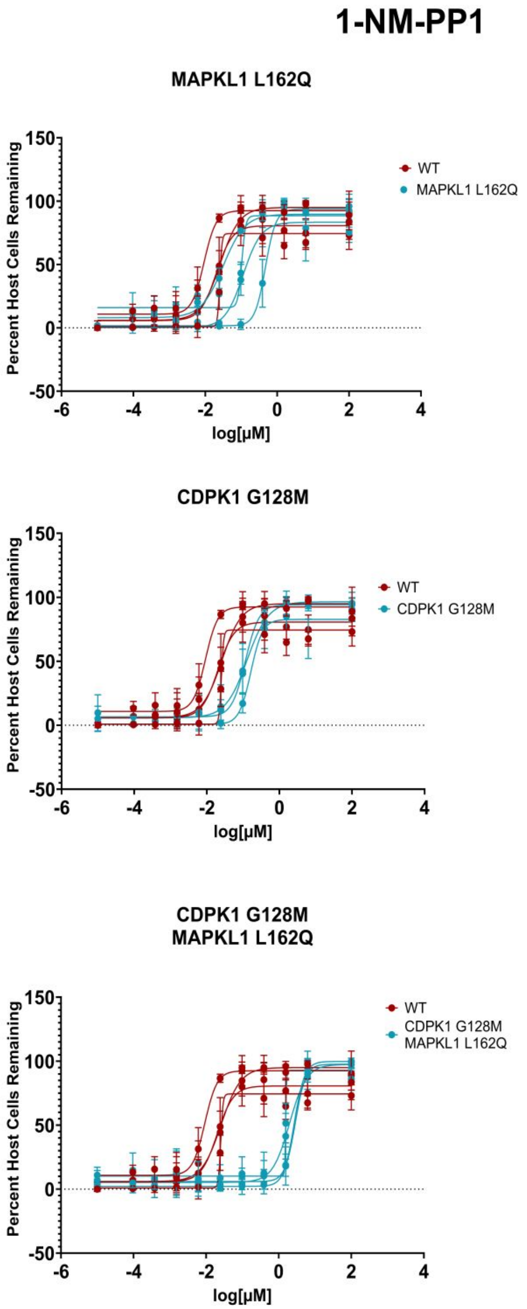

Figure S2: EC_{50} (50% effective concentration) of BKIs tested in this study against strains of *T. gondii* with amino acid substitutions in *Tg*MAPKL1, *Tg*CDPK1, and both were introduced with site-directed mutagenesis compared to the parent strain. N≥3 for each drug strain combination. EC_{50} values in Table 2 in the body of the manuscript. Error bars represent standard deviation between replicates within each trial. WT indicates parental strain.

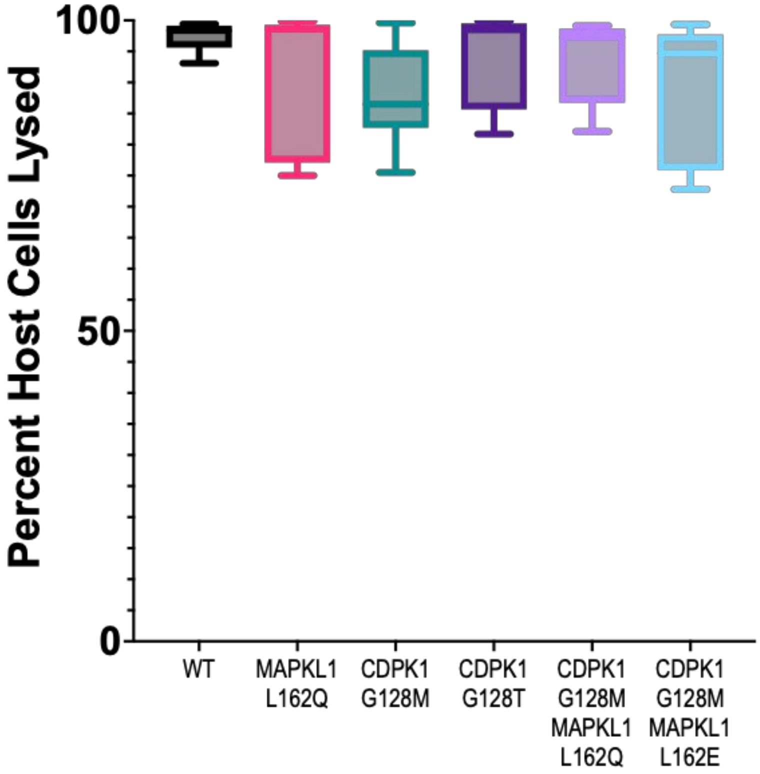

Figure S3: Percent of fibroblast cells lysed after 5 days by BKI-1748 resistant strains that were created by site-directed mutagenesis.

Clone 1A	Chromosome	Position	Quality score	Sequence Ontology	Nucleotide	Protein	ToxoDB Product Description
TGGT1_224160	TGGT1_chrX	3203183	53.6407	missense	6134G>A	Cys2045Tyr	hypothetical protein
TGGT1_216590	TGGT1_chrXI	5988029	119.229	missense	737C>T	Ala246Val	HEAT repeat-containing protein
TGGT1_229230	TGGT1_chrVIII	103068	319.523	missense	766T>C	Ser256Pro	hypothetical protein
TGGT1_229020	TGGT1_chrVIII	33034	50.1457	missense	166A>C	Asn56His	putative cell-cycle-associated protein kinase CDK
TGGT1_305120	TGGT1_chrIX	5006278	718.956	missense	436T>C	Tyr146His	transporter, solute:sodium symporter (SSS) family protein
TGGT1_269020	TGGT1_chrVIII	6131213	114.192	missense	150A>C	Lys50Asn	hypothetical protein
TGGT1_226810	TGGT1_chrX	1251540	52.7199	missense	18783A>C	Lys6261Asn	histone lysine methyltransferase SET1
TGGT1_269100	TGGT1_chrVIII	6059113	54.4053	missense	982C>A	His328Asn	hypothetical protein
TGGT1_263610	TGGT1_chrVIIb	407909	68.722	missense	1334T>C	Phe445Ser	hypothetical protein
TGGT1_235420	TGGT1_chrX	4751025	492.788	missense	4874T>G	Leu1625Arg	hypothetical protein
TGGT1_312570	TGGT1_chrXI	2755695	468.705	missense	485T>A	Leu162Gln	CMGC kinase, MAPK family (ERK) MAPK-1
TGGT1_316120	TGGT1_chrXI	5085521	459.328	missense	1835A>G	Glu612Gly	hypothetical protein
TGGT1_278815	TGGT1_chrXII	5769272	81.1257	missense	1975G>A	Glu659Lys	hypothetical protein
TGGT1_297350	TGGT1_chrII	1686461	351.388	missense	1003G>A	Ala335Thr	hypothetical protein
TGGT1_297790	TGGT1_chrII	1979032	65.6385	missense	719T>G	Leu240Arg	hypothetical protein
TGGT1_244500	TGGT1_chrVI	3234544	308.459	missense	176A>G	His59Arg	Tubulin-tyrosine ligase family protein
**Clone 1B**							
TGGT1_229230	TGGT1_chrVIII	103068	380.616	missense	766T>C	Ser256Pro	hypothetical protein
TGGT1_305120	TGGT1_chrIX	5006278	840.518	missense	436T>C	Tyr146His	transporter, solute:sodium symporter (SSS) family protein
TGGT1_269020	TGGT1_chrVIII	6131213	55.0473	missense	150A>C	Lys50Asn	hypothetical protein
TGGT1_226950	TGGT1_chrX	1111614	80.8746	missense	3059T>G	Leu1020Arg	IgA-specific metalloendopeptidase
TGGT1_226810	TGGT1_chrX	1251540	69.9123	missense	18783A>C	Lys6261Asn	histone lysine methyltransferase SET1
TGGT1_235420	TGGT1_chrX	4751025	611.441	missense	4874T>G	Leu1625Arg	hypothetical protein
TGGT1_312570	TGGT1_chrXI	2755695	818.221	missense	485T>A	Leu162Gln	CMGC kinase, MAPK family (ERK) MAPK-1
TGGT1_311250	TGGT1_chrXI	1878963	55.0634	missense	3289G>A	Asp1097Asn	hypothetical protein
TGGT1_220290	TGGT1_chrV	170935	66.8724	missense	3235A>G	Ile1079Val	hypothetical protein
TGGT1_297350	TGGT1_chrII	1686461	761.808	missense	1003G>A	Ala335Thr	hypothetical protein
TGGT1_244500	TGGT1_chrVI	3234544	700.576	missense	176A>G	His59Arg	Tubulin-tyrosine ligase family protein
**Clone 2**							
TGGT1_229230	TGGT1_chrVIII	103068	582.595	missense	766T>C	Ser256Pro	hypothetical protein
TGGT1_305120	TGGT1_chrIX	5006278	684.342	missense	436T>C	Tyr146His	transporter, solute:sodium symporter (SSS) family protein
TGGT1_260340	TGGT1_chrVIIb	2318544	153.835	missense	431C>T	Pro144Leu	DNL zinc finger protein
TGGT1_239795	TGGT1_chrVI	913982	60.3403	missense	271C>T	Arg91Trp	hypothetical protein
TGGT1_263610	TGGT1_chrVIIb	407950	121.926	missense	1375C>A	Pro459Thr	hypothetical protein
TGGT1_235420	TGGT1_chrX	4751025	175.305	missense	4874T>G	Leu1625Arg	hypothetical protein
TGGT1_312570	TGGT1_chrXI	2755695	547.482	missense	485T>A	Leu162Gln	CMGC kinase, MAPK family (ERK) MAPK-1
TGGT1_316120	TGGT1_chrXI	5085521	617.498	missense	1835A>G	Glu612Gly	hypothetical protein

TGGT1_297350	TGGT1_chrII	1686461	1048.34	missense	1003G>A	Ala335Thr	hypothetical protein
TGGT1_237480	TGGT1_chrX	5798884	50.7404	missense	3982G>A	Glu1328Lys	BRCA1 C Terminus (BRCT) domain-containing Protein
TGGT1_244500	TGGT1_chrVI	3234544	617.941	missense	176A>G	His59Arg	Tubulin-tyrosine ligase family protein
**Clone 3A**							
TGGT1_247450	TGGT1_chrXII	3283101	1245.07	missense	5991C>G	Phe1997Leu	hypothetical protein
TGGT1_280480	TGGT1_chrVIIa	353328	1528.99	missense	647T>C	Met216Thr	EF hand domain-containing protein
TGGT1_263610	TGGT1_chrVIIb	407909	56.8703	missense	1334T>C	Phe445Ser	hypothetical protein
TGGT1_263610	TGGT1_chrVIIb	407917	102.278	missense	1342C>A	Pro448Thr	hypothetical protein
TGGT1_312570	TGGT1_chrXI	2755054	1425.05	missense	571T>A	Ser191Thr	CMGC kinase, MAPK family (ERK) MAPK-1
TGGT1_232380	TGGT1_chrVIII	2061726	1228.97	missense	1015A>G	Ser339Gly	WD domain, G-beta repeat-containing protein
TGGT1_316730	TGGT1_chrXI	5488072	106.879	missense	4585T>A	Ser1529Thr	Sma protein
TGGT1_253750	TGGT1_chrIII	1138897	1100.76	missense	13331A>T	Gln4444Leu	PLU-1 family protein
TGGT1_227360	TGGT1_chrX	902650	1007.29	missense	310A>G	Thr104Ala	ribosomal protein RPL3
TGGT1_226390	TGGT1_chrX	1574594	55.5691	missense	1093C>T	Arg365Cys	hypothetical protein
**Clone 3B**							
TGGT1_462965	TGGT1_chrVIIa	959300	1394.36	missense	4604A>G	Asp1535Gly	formin domain-containing protein
TGGT1_205050	TGGT1_chrVIIa	1564289	1359.27	missense	685T>C	Tyr229His	hypothetical protein
TGGT1_236670B	TGGT1_chrX	5382618	78.1638	missense	836C>A	Thr279Lys	hypothetical protein
TGGT1_246910	TGGT1_chrXII	2945913	1194.57	stop_gained	776G>A	Trp259*	putative histone lysine methyltransferase, SET
TGGT1_203135	TGGT1_chrVIIa	2668047	1074.64	missense	7426C>A	Pro2476Thr	dynein heavy chain family protein
TGGT1_312570	TGGT1_chrXI	2755668	1156.64	missense	512T>C	Ile171Thr	CMGC kinase, MAPK family (ERK) MAPK-1
TGGT1_209270	TGGT1_chrIb	1178284	1309.04	missense	4333T>C	Phe1445Leu	hypothetical protein
TGGT1_220110	TGGT1_chrV	38483	1128.8	missense	3677T>C	Val1226Ala	hypothetical protein
**Clone 3C**							
TGGT1_462965	TGGT1_chrVIIa	959300	694.752	missense	4604A>G	Asp1535Gly	formin domain-containing protein
TGGT1_205050	TGGT1_chrVIIa	1564289	1069.03	missense	685T>C	Tyr229His	hypothetical protein
TGGT1_289280	TGGT1_chrIX	2944313	63.6678	missense	3243C>A	Asn1081Lys	hypothetical protein
TGGT1_246910	TGGT1_chrXII	2945913	1317.89	Stop gained	776G>A	Trp259*	putative histone lysine methyltransferase, SET
TGGT1_203135	TGGT1_chrVIIa	2668047	967.816	missense	7426C>A	Pro2476Thr	dynein heavy chain family protein
TGGT1_312570	TGGT1_chrXI	2755668	838.226	missense	512T>C	Ile171Thr	CMGC kinase, MAPK family (ERK) MAPK-1
TGGT1_310720B	TGGT1_chrXI	1507009	62.839	missense	31G>C	Ala11Pro	hypothetical protein
TGGT1_249510	TGGT1_chrXII	4519780	50.7404	missense	548C>T	Pro183Leu	hypothetical protein
TGGT1_209270	TGGT1_chrIb	1178284	541.471	missense	4333T>C	Phe1445Leu	hypothetical protein
TGGT1_220110	TGGT1_chrV	38483	1516.31	missense	3677T>C	Val1226Ala	hypothetical protein
TGGT1_278518	TGGT1_chrXII	5961769	92.4619	missense	1484C>T	Ala495Val	N-acetylgalactosaminyl transferase

Table S1: Genes containing nonsynonymous single nucleotide variants found in the resistant clones compared to their parental strain. *Tg*MAPKL1 is highlighted in red. Genes identified with ToxoDB gene ID numbers.

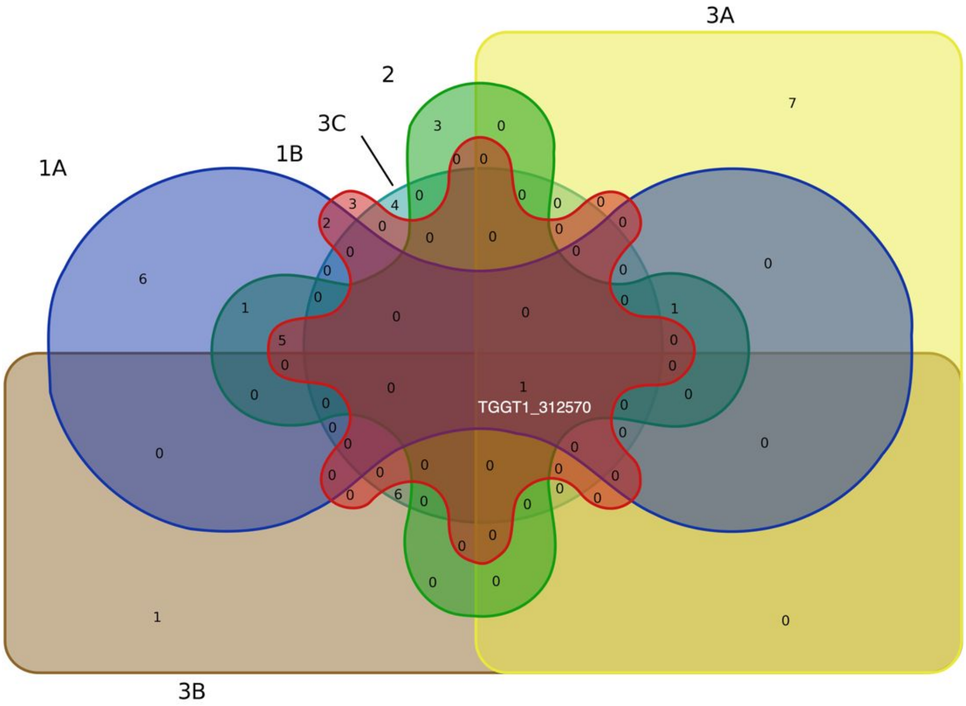

Figure S4: Venn diagram of Supplemental Table 1 overlap of single nucleotide variant between clones. TGGT1_312570 is the single gene where all clones overlap.

CDPK1_mut1_Thr	GCGAGGTGCAGTTGCTGAAGCAGCTGGACC ACCCCAACATCATGAAGCTGTATGAATTCTT CGAGGACAAAGGCTACTTCTACCTCGTCACT GAAGTGTACACGGGAGGCGAGTTGTTCGAC GAGATCATTTCCCGCAAG
CDPK1 mut1 Met	GCGAGGTGCAGTTGCTGAAGCAGCTGGACC ACCCCAACATCATGAAGCTGTATGAATTCTT CGAGGACAAAGGCTACTTCTACCTCGTCAT GGAAGTGTACACGGGAGGCGAGTTGTTCGA CGAGATCATTTCCCGCAAG
CDPK1 mut1	AAAGGCTACTTCTACCTCGT
MAPK mut1	TTTCGACGAGATTGATGATATTTTCATGTTT CAACTTCTTCTGAATCTTGATCTCTCTGTAG ATGCGTTTGGCGTCGATCAAATCACGAAAC AAATCACCGATCTTCTTCACCGCAACTTTCT TGTT
MAPK mut1	CAGAGAGATCAAGATTCTGA

Table S2: Oligonucleotides
